# The activity of the endocannabinoid metabolising enzyme fatty acid amide hydrolase in subcutaneous adipocytes correlates with BMI in metabolically healthy humans

**DOI:** 10.1186/1476-511X-10-129

**Published:** 2011-08-04

**Authors:** Jemma C Cable, Garry D Tan, Stephen PH Alexander, Saoirse E O'Sullivan

**Affiliations:** 1 School of Graduate Entry Medicine and Health, University of Nottingham, Derby, DE22 3DT, UK; 2 School of Biomedical Sciences, University of Nottingham, Nottingham, NG7 2UH, UK

**Keywords:** Endocannabinoid system, Fatty acid amide hydrolase, Monoacylglycerol lipase, Human, Adipocytes, BMI, Insulin, Glucose

## Abstract

**Background:**

The endocannabinoid system (ECS) is a ubiquitously expressed signalling system, with involvement in lipid metabolism and obesity. There are reported changes in obesity of blood concentrations of the endocannabinoids anandamide (AEA) and 2-arachidonoylglcyerol (2-AG), and of adipose tissue expression levels of the two key catabolic enzymes of the ECS, fatty acid amide hydrolase (FAAH) and monoacylglycerol lipase (MGL). Surprisingly, however, the activities of these enzymes have not been assayed in conditions of increasing adiposity. The aim of the current study was to investigate whether FAAH and MGL activities in human subcutaneous adipocytes are affected by body mass index (BMI), or other markers of adiposity and metabolism.

**Methods:**

Subcutaneous abdominal mature adipocytes, fasting blood samples and anthropometric measurements were obtained from 28 metabolically healthy subjects representing a range of BMIs. FAAH and MGL activities were assayed in mature adipocytes using radiolabelled substrates. Serum glucose, insulin and adipokines were determined using ELISAs.

**Results:**

MGL activity showed no relationship with BMI or other adiposity indices, metabolic markers (fasting serum insulin or glucose) or serum adipokine levels (adiponectin, leptin or resistin). In contrast, FAAH activity in subcutaneous adipocytes correlated positively with BMI and waist circumference, but not with skinfold thickness, metabolic markers or serum adipokine levels.

**Conclusions:**

In this study, novel evidence is provided that FAAH activity in subcutaneous mature adipocytes increases with BMI, whereas MGL activity does not. These findings support the hypothesis that some components of the ECS are upregulated with increasing adiposity in humans, and that AEA and 2-AG may be regulated differently.

## Introduction

The endocannabinoid system (ECS) is expressed in most human tissues and comprises the endocannabinoids, their receptors and the enzymes required for their synthesis and degradation. The two best characterised endocannabinoids are *N*-arachidonoylethanolamide (anandamide, AEA) [1] and 2-arachidonoylglycerol (2-AG) [2,3]. Fatty acid amide hydrolase (FAAH) is responsible for the majority of AEA hydrolysis and also accounts for a minor amount of 2-AG inactivation [4,5], but this is predominantly catalysed by monoacylglycerol lipase (MGL) [4].

The ECS is present in human adipocytes [6], although relatively little is understood of its role in adipose tissue. Both AEA and 2-AG have been identified in isolated adipocytes from human visceral adipose tissue [7], while human subcutaneous adipocytes have been shown to synthesise both endocannabinoids [8]. It has also been demonstrated, in a murine preadipocyte cell line, that induction of adipogenesis leads to a significant increase in intracellular levels of AEA and 2-AG, and that 2-AG concentrations remain high in mature adipocytes [7,9]. In addition, AEA has been shown to induce differentiation of murine preadipocytes, possibly by direct activation of PPARγ [10]. With regard to the expression of ECS-related enzymes, mature adipocytes contain higher levels of FAAH mRNA than preadipocytes [11], indicating that the ECS may have an important role in functional adipocytes. A role for the ECS in human adipocytes is further supported by the presence of the two major endocannabinoid receptors, CB_1 _and CB_2 _[12]. However, the precise role of the ECS in adipocytes is still a matter for investigation (for review see [13]).

It has been suggested that overall ECS tone is increased in human obesity on the basis that reports of plasma levels of AEA [11,14] and 2-AG [15] correlate positively with BMI. Circulating 2-AG levels correlate with waist circumference [11] and more in depth analysis has shown that the most significant rise in 2-AG occurs in those with visceral obesity [14,15]. Additionally, weight loss in obese men has been shown to reduce plasma levels of both AEA and 2-AG [16]. However, the relative expression levels of components of the ECS in adipose tissue in obese compared to lean humans have yet to be confirmed.

Levels of FAAH mRNA expression in human adipose tissue have been measured by multiple laboratories and conflicting findings have been reported. In some studies, FAAH mRNA is reported to be higher in the subcutaneous adipose tissue of obese compared to lean subjects [17,18], whereas other studies report FAAH mRNA to be decreased [11,14,19]. According to one study, hyperinsulinaemia increased FAAH mRNA in the subcutaneous abdominal adipose tissue in lean, but not in obese, subjects leading the authors to suggest that the chronic hyperinsulinaemia often present in obese humans could contribute to FAAH upregulation in adipose tissue [18]. There are no obvious reasons as to why discrepancies have been reported with regard to FAAH expression levels in adipose tissue in obesity. The techniques used in these studies appear to have been similar, as do the subjects sampled, although females are represented more than males in the studies showing FAAH to be downregulated in obesity, and males are a larger proportion of the results showing FAAH to be upregulated. A further connection between FAAH and obesity has been identified via a missense mutation in the FAAH gene, which occurs in 3.6-10.8% of the population (depending on ethnicity) and is associated with obesity [20].

Most of the above studies have compared lean and obese subjects, and all have reported mRNA levels of FAAH without reference to final protein levels or activity. To the best of our knowledge, to date, there is only one published study on the enzyme activity of FAAH in human adipose tissue, and this was performed only to confirm its presence [21]. In order to increase our understanding of the role of FAAH in human adipocytes, and whether this alters with BMI, it is important to investigate FAAH activity, and thus endocannabinoid degradation, in isolated adipocytes.

MGL expression in human adipose tissue has not yet been extensively investigated with regard to obesity. In one study, MGL mRNA in omental and subcutaneous adipose tissue was compared between distinct cohorts of lean and obese humans and it was found that in omental adipose, MGL mRNA was decreased with obesity, but that obesity had no effect on MGL expression in abdominal subcutaneous adipose tissue [19]. However, in another study, MGL mRNA was found to be upregulated in the abdominal subcutaneous and omental adipose tissue of obese subjects [17]. Knowing that plasma 2-AG is particularly increased in obesity [15], and the speculation that 2-AG secretion from adipocytes may contribute to this, it is important to establish the effects of obesity on MGL activity in adipocytes.

The current study was therefore designed to investigate the activity of FAAH and MGL in adipocytes taken from healthy human volunteers representing a continuous range of BMIs from normal to obese. Assays were undertaken in mature adipocytes isolated from human subcutaneous adipose tissue to exclude interference from other cells in adipose tissue such as preadipocytes or immune cells (other studies measured enzyme expression in the entire adipose tissue sample [11,14,17-19,21]). In addition, a number of obesity-related physical and metabolic parameters were investigated. The primary aim of the study was to investigate the effects of BMI on FAAH and MGL activity in human adipocytes. A secondary objective was to examine whether a relationship exists between these enzyme activities and various measures of adiposity and metabolism.

## Materials and methods

### Subjects

The study was approved by the University of Nottingham Medical School Ethics Committee. 28 volunteers were recruited from within the University of Nottingham. Written informed consent was obtained and exclusion criteria included smoking, hypertension and known metabolic disease. All subjects reported a stable weight in the three month period preceding the biopsy.

### Anthropometric measurements

Blood pressure was measured with subjects rested and in the supine position. Waist circumference was measured at the midpoint between the iliac crest and costal margin, and hip circumference was taken at the widest point around the hips. Neck circumference was measured at the level of the cricothyroid cartilage and arm circumference was measured at the midpoint between the shoulder and elbow. Skinfold thickness was measured using Harpenden calipers at the following anatomical sites: tricep, bicep, subscapular, iliac crest, abdominal, chest and midaxilla [22]. The values obtained from each were summed to give an indication of the amount of subcutaneous body fat for each subject.

### Adipose and blood sampling

Subjects were asked to fast for at least 12 hours prior to the adipose tissue biopsy. The subcutaneous abdominal adipose biopsies were aspirated using a needle under local anaesthetic with 1% lidocaine. Venous blood samples were obtained and serum was separated and stored at -80°C.

### Isolation and homogenisation of mature adipocytes

The method used to obtain mature adipocytes was adapted from that described by Rodbell [23]. The adipose samples were immediately added to an equal volume of type II collagenase in phosphate buffered saline (PBS; 1 mg.ml^-1^; Sigma-Aldrich, UK) and allowed to digest at 37°C for 45 minutes. The samples were then washed twice in PBS using centrifugation (500 × *g*, 2 minutes) to separate the mature adipocytes which formed a floating layer. The isolated adipocytes were stored at -80°C until homogenisation.

Cells were homogenised in TE buffer (50 mM Tris, 1 mM EDTA, pH 7.4) using a hand-held glass homogeniser on ice. The homogenates were centrifuged (18,000 × *g*, 10 minutes) and the supernatant layer then removed and spun again (20,000 × *g*, 30 minutes). The supernatant layer from this step was then stored at -80°C as the cytosolic fraction. The cellular pellet was homogenised in PBS (10 mM phosphate, 2.7 mM potassium chloride, 137 mM sodium chloride, pH 7.4), centrifuged (20,000 × *g*, 30 minutes), re-suspended and stored at -80°C.

### Enzyme activity assays

Enzyme assays were conducted essentially as described by Boldrup *et al*., 2004 [24]. In brief, the particulate fraction of the adipocyte homogenates was assayed in duplicate for FAAH activity. Sample aliquots were diluted in TE buffer containing fatty acid free albumin at 1 mg.ml^-1 ^(pH 7.4) and pre-incubated at 37°C for 10 minutes with the FAAH inhibitor URB597 (1 μM, Sigma Chemical Company, UK), or vehicle. [^3^H]-AEA (2 μM, American Radiolabelled Chemicals, USA) was added and the samples were incubated at 37°C for 30 minutes. Activated charcoal (2 volumes, 10% w/v in 0.5 M HCl) was used to stop the reaction. After brief centrifugation, an aliquot of each supernatant layer was taken for scintillation counting. Tubes without homogenate were run in parallel and used to establish blank values. In all cases, activity in the presence of URB597 was no different from blanks.

The cytosolic fraction of the adipocyte homogenates was assayed in duplicate for MGL activity using a similar method as above, substituting a MGL inhibitor, methylarachidonylfluorophosphonate (MAFP, 1 μM, Sigma Chemical Company, UK), and 2-oleoyl-[^3^H]-glycerol (2-OG, 100 μM, American Radiolabelled Chemicals, USA). In this assay the samples were incubated at 37°C for 15 minutes. In all cases, activity in the presence of MAFP was no different from blanks.

### Blood serum analysis

Aliquots of blood serum were thawed immediately prior to testing, and glucose and insulin assays were performed within 6 months of sample collection. Serum glucose concentrations were determined using the YSI 2300 STAT PLUS glucose and lactate analyser (YSI Life Sciences, USA). Insulin concentrations of the serum samples were measured using a commercially available ELISA kit (Mercodia, Sweden). The homeostatic model assessment (HOMA2-%S) figures were calculated using the HOMA2 model (http://www.dtu.ox.ac.uk). Plasma adiponectin, leptin and resistin concentrations were measured within 18 months of sample collection via commercially available sandwich ELISAs (DuoSets from R&D, USA). All samples were tested in duplicate.

### Statistical analysis

GraphPad Prism (California, USA) software was used to analyse all of the data, using linear regression to report the Pearson correlation coefficient.

## Results

### Subject demographics

The results of this study were obtained from metabolically healthy humans ranging in BMI from 19.1-33.8 kg.m^-2^. Physiological data of these subjects are given in Table 1. All subjects had a fasting blood glucose concentration of < 5.6 mmol.L^-1 ^on the day of biopsy. Correlation studies confirmed that subjects reported in this study follow well-documented findings in the literature; for example, BMI correlated with MAP (r^2 ^= 0.22, *P *< 0.05), waist (r^2 ^= 0.69, *P *< 0.001), and the sum of the skinfold thicknesses (r^2 ^= 0.39, *P *< 0.001).

**Table 1:** Anthropometric and physiological data of study subjects

Male:female	14:14
Age (years)	20-48 31 ± 8.7

BMI (kg.m^-2^)	19.1-33.8 24.2 ± 3.4

Systolic blood pressure (mmHg)	104-135 120.5 ± 8.8

Diastolic blood pressure (mmHg)	60-91 73.4 ± 7.2

Mean arterial pressure (mmHg)	77-106 89.1 ± 6.2

Waist circumference (cm)	69-114 82.2 ± 10.7

Sum of 7-point skinfolds (mm)	63-173 99.9 ± 25.5

Fasting serum glucose (mmol.L^-1^)	4.4-5.6 5.1 ± 0.3

Fasting serum insulin (pmol.L^-1^)	12.5-91.0 40.3 ± 21.9

HOMA2-%S	59.4-361.5 165.0 ± 79.2

Serum adiponectin (μg.ml^-1^)	3.55-21.28 10.93 ± 5.0

Serum leptin (ng.ml^-1^)	0.34-36.53 8.5 ± 15.4

Serum resistin (ng.ml^-1^)	5.38-32.07 11.49 ± 7.2

Data are expressed as range, and as mean ± S.D. BMI, body mass index; HOMA2-%S, homeostatic model assessment for insulin sensitivity.

### Endocannabinoid-metabolising enzyme activities and BMI

**Figure 1:**
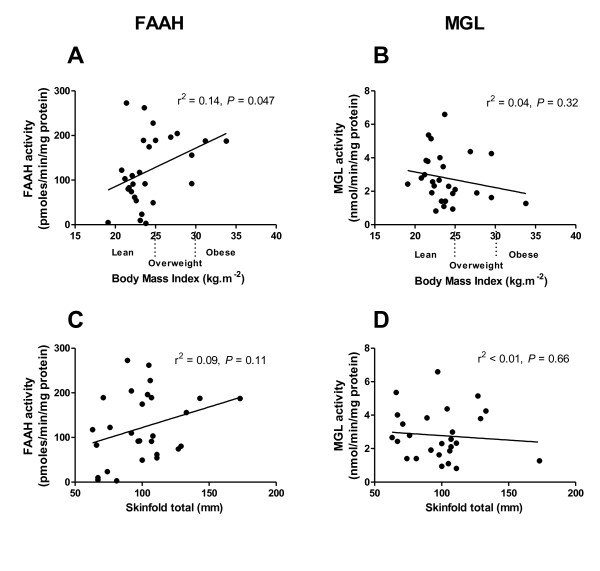
**FAAH and MGL activities in abdominal subcutaneous adipocytes with relation to BMI (A and B) and the sum of skinfold thicknesses at 7 sites (C and D)**. Analysed using linear regression.

In these metabolically healthy subjects, MGL activity in subcutaneous mature adipocytes did not correlate with BMI (r^2 ^= 0.04, *P *= 0.32; Figure 1B), the sum of all 7 skinfold thicknesses (r^2 ^< 0.01, *P *= 0.66; Figure 1D) or with skinfold thickness at each individual site measured (see Table 2). In contrast, FAAH activity in subcutaneous mature adipocytes correlated positively with BMI (r^2 ^= 0.14, *P *= 0.047; Figure 1A). The sum of the skinfold thicknesses did not correlate with FAAH activity (r^2 ^= 0.09, *P *= 0.11; Figure 1C). There was similarly no correlation between FAAH activity and any of the individual skinfold thicknesses measured (Table 2) with FAAH activity.

**Table 2:** Correlation studies between FAAH and MGL activities in subcutaneous adipocytes and skinfold thickness at various sites (Pearson correlation coefficients)

		Tricep	Bicep	Abdominal	Iliac crest	Subscapular	Chest	Midaxilla
**FAAH**	r^2^	< 0.01	0.04	0.11	0.06	0.08	0.08	0.12
	
**activity**	*P*	0.76	0.33	0.09	0.20	0.14	0.14	0.07

**MGL**	r^2^	0.02	0.01	0.03	< 0.01	0.02	< 0.01	0.04
	
**activity**	*P*	0.53	0.59	0.43	0.88	0.50	0.97	0.34

### Endocannabinoid-metabolising enzyme activities and circumferences

**Figure 2:**
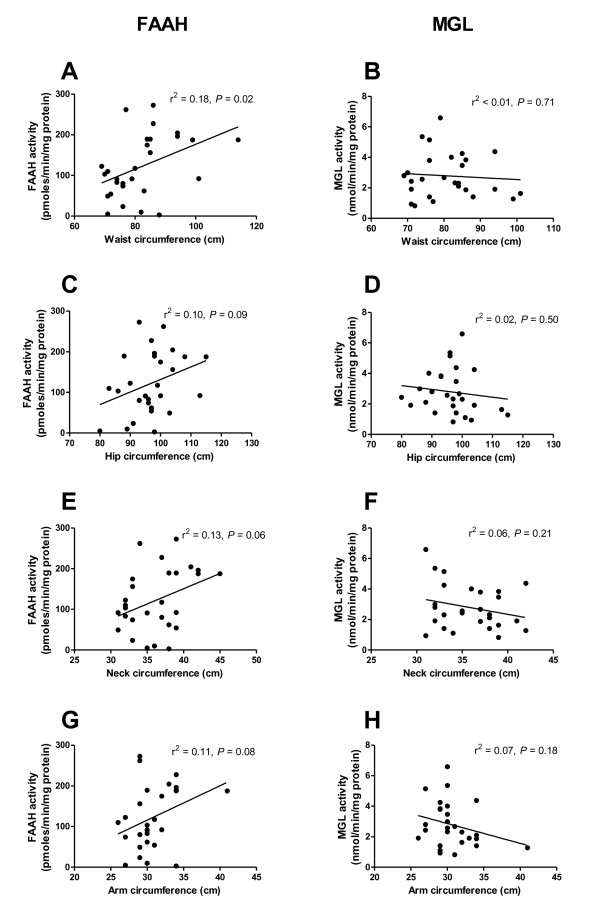
**FAAH and MGL activities in abdominal subcutaneous adipocytes and circumferences of the waist (A and B), hip (C and D), neck (E and F) and arm (G and H)**. Analysed using linear regression.

FAAH activity was found to correlate positively with waist circumference (r^2 ^= 0.18, *P *= 0.023; Figure 2A). FAAH activity showed a trend towards a positive correlation (all *P *< 0.1) with hip circumference (r^2 ^= 0.10, *P *= 0.094; Figure 2C), neck circumference (r^2 ^= 0.13, *P *= 0.055; Figure 2E) and arm circumference (r^2 ^= 0.11, *P *= 0.080; Figure 2G). In contrast, MGL activity did not correlate with waist circumference (r^2 ^< 0.01, *P *= 0.71; Figure 2B), hip circumference (r^2 ^= 0.02, *P *= 0.50; Figure 2D), neck circumference (r^2 ^= 0.06, *P *= 0.21; Figure 2F) or arm circumference (r^2 ^= 0.07, *P *= 0.18; Figure 2H).

### Endocannabinoid-metabolising enzyme activities and glycaemic markers

**Figure 3:**
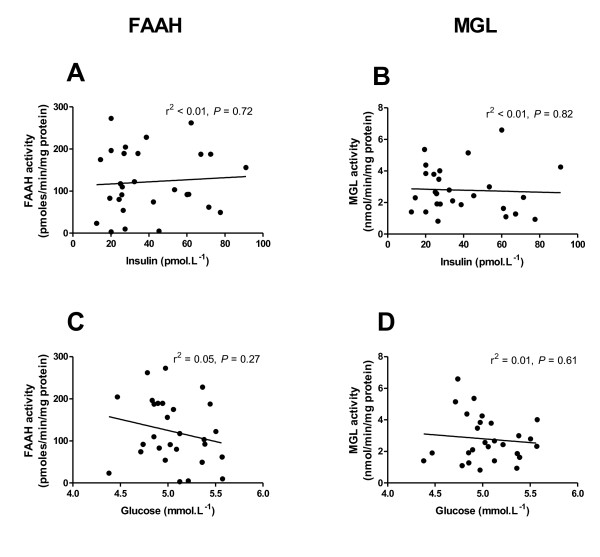
**FAAH and MGL activities in abdominal subcutaneous adipocytes and blood concentrations of insulin (A and B) and glucose (C and D) in fasted subjects**. Analysed using linear regression.

Although the subjects included in this study were considered metabolically healthy, there was a range of fasting serum glucose (4.4-5.6 mmol.L^-1^) and insulin (12.5-91.0 pmol.L^-1^) values. Neither fasting insulin (r^2 ^< 0.01, *P *= 0.72; Figure 3A) nor glucose (r^2 ^= 0.05, *P *= 0.27; Figure 3C) showed any relationship with FAAH activity in mature abdominal subcutaneous adipocytes. This was also true for HOMA2-%S (r^2 ^< 0.01, *P *= 0.78). Similarly, MGL activity did not correlate with fasting serum concentrations of insulin (r^2 ^< 0.01, *P *= 0.82; Figure 3B), glucose (r^2 ^= 0.01, *P *= 0.61; Figure 3D), or with HOMA2-%S (r^2 ^= 0.01, *P *= 0.56).

### Endocannabinoid-metabolising enzyme activities and serum adipokines

**Figure 4:**
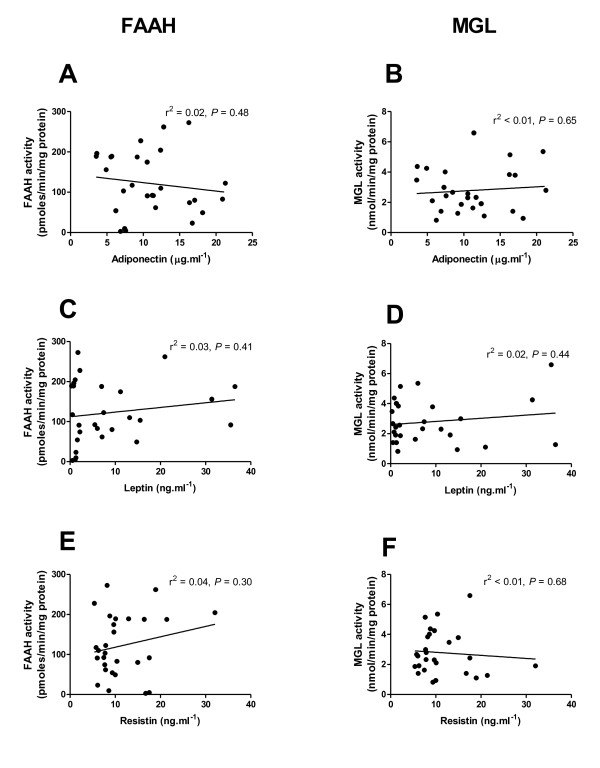
**FAAH and MGL activities in abdominal subcutaneous adipocytes and blood concentrations of adiponectin (A and B), leptin (C and D) and resistin (E and F) in fasted subjects**. Analysed using linear regression.

Fasting serum concentrations of adiponectin (r^2 ^= 0.02, *P *= 0.48; Figure 4A), leptin (r^2 ^= 0.03, *P *= 0.41; Figure 4C) and resistin (r^2 ^= 0.04, *P *= 0.298; Figure 4E) did not correlate with FAAH activity in subcutaneous adipocytes. MGL activity also failed to correlate with fasting serum concentrations of adiponectin (r^2 ^< 0.01, *P *= 0.65; Figure 4B), leptin (r^2 ^= 0.02, *P *= 0.44; Figure 4D) or resistin (r^2 ^< 0.01, *P *= 0.68; Figure 4F).

## Discussion

The principal aim of the current study was to investigate whether the activities of FAAH and MGL, two key catabolic enzymes of the ECS, are altered with increasing BMI. In measuring the activities of the enzymes, rather than mRNA, we are able to present novel data that FAAH activity in human subcutaneous mature adipocytes increases with BMI and waist circumference. In contrast there is no relationship between MGL activity and BMI or the other adiposity markers measured. Neither FAAH nor MGL activity correlates with fasting serum concentrations of insulin, glucose or various adipokines in these healthy volunteers.

In several published studies, FAAH mRNA levels in adipose tissue have been compared between lean and obese humans, and there is conflict over whether FAAH is up- or down-regulated in adipose tissue in obesity [11,14,17-19]. In order to investigate this further, we measured FAAH activity in our population, as mRNA levels do not always accurately reflect final protein levels [25]. We found that in the abdominal subcutaneous mature adipocytes of metabolically healthy people, FAAH activity increases with BMI and waist circumference. This finding is in agreement with studies showing increased FAAH mRNA in the subcutaneous adipose of obese individuals compared to lean [17,18], but at odds with others [11,14,19]. The reasons for the apparently discrepant results are currently unclear, but two of these studies used entirely female samples [11,19], and the other studied surgical patients [14]. It should also be noted that our results are not necessarily directly comparable to previous findings, given that previous studies have used whole adipose tissue homogenates and we have specifically studied mature adipocytes. This is important when considering the ECS in a metabolic context, as mature adipocytes are the adipose cells involved in energy homeostasis, but mature adipocytes account for only half of the cells in adipose tissue. In particular, it has been shown that macrophages have significant FAAH expression [26]. In addition to this, we have used subjects representing a continuous range of BMIs in this study, as opposed to the discrete cohorts of lean and obese subjects used in many studies. This has allowed the inclusion of data on people with a BMI between 25 and 30, which is a group that has not been described previously. The increase of FAAH activity with BMI that we observed may be metabolically protective, as missense mutations in the FAAH gene have been associated with an unfavourable metabolic profile in obese subjects [27].

The ECS is involved in the regulation of metabolism and feeding in many human tissues, and some of its components, particularly CB_1_, AEA and 2-AG, have been found to be upregulated in obesity [28,29]. An increase in FAAH activity in adipocytes with increasing BMI may simply be part of this general upregulation of ECS tone in obesity. If both the synthesis and degradation of endocannabinoids are upregulated similarly, this combination would not be predicted to affect endocannabinoid signalling and the functional effects of endocannabinoids within the adipocyte. This hypothesis is supported by the recent finding that AEA levels in subcutaneous adipose tissue are not different between lean and non-diabetic obese humans [30]. Alternatively, FAAH may be upregulated in isolation. This could reduce AEA signalling at CB_1 _and CB_2 _receptors, and at intracellular targets such as TRPV1 and PPARs, potentially reducing CB_1 _mediated glucose uptake, lipogenesis and adipogenesis [13].

It is known that in humans of the same BMI, visceral adipose tissue accumulation confers greater metabolic and cardiovascular risk than excess subcutaneous adipose tissue [31]. With this in mind, we measured skinfold thickness at various anatomical sites to give an indication of fat distribution in the subjects studied. In addition to the correlation between FAAH activity and waist circumference, we found a non-significant positive relationship with hip circumference, neck circumference and two central skinfold thicknesses (abdominal and midaxillary), but not with the two peripheral skinfolds measured (bicep and tricep). This is in keeping with results showing that visceral obesity is a better predictor of circulating 2-AG levels than non-specific obesity [14] and may support the notion that central adipose accumulation is more significant in relation to the ECS than the amount of subcutaneous adipose tissue.

In one study showing chronic FAAH upregulation in the subcutaneous adipose tissue of obese humans, it was suggested that hyperinsulinaemia may cause this upregulation, as similarly high FAAH expression was induced in healthy lean humans using the euglycaemic hyperinsulinaemic clamp method [18]. Considering this, we investigated FAAH activity in comparison with fasting serum levels of insulin and glucose, and HOMA2-%S (an estimation of insulin sensitivity). In this sample of healthy volunteers, there was no correlation between FAAH activity in subcutaneous adipocytes and fasting serum concentrations of glucose or insulin, or HOMA2-%S, although it should be noted that all subjects had fasting serum levels of these parameters within normal ranges. In order to investigate further a relationship between insulin or glucose levels and FAAH activity in adipocytes, further studies need to be conducted to include subjects with poor glycaemic control.

Serum adipokine levels are known to be dysregulated in obesity, with downregulation of adiponectin and upregulation of leptin and resistin. As yet, few *in vivo *studies have investigated adipokine levels with regard to the ECS. We found that in healthy humans, FAAH activity in adipocytes is not correlated with fasting serum concentrations of adiponectin, leptin or resistin. It has been shown that a FAAH missense mutation is associated with both lower serum adiponectin concentrations in diabetic patients [32], and higher serum leptin levels after weight loss in obese humans [27], although the systemic nature of the mutation makes it unclear as to the extent of adipocyte involvement in these changes. *In vitro*, leptin has been shown to increase FAAH activity in T lymphocytes [33], although not in neuroblastoma cells [34], and our results suggest that *in vivo *leptin does not significantly affect FAAH activity in adipocytes.

MGL has a primary role in lipid metabolism, specifically in the hydrolysis of monoacylglycerols to release glycerol and fatty acids that are subsequently transported out of the adipocyte. This explains the relatively high activity of MGL (~300 fold) compared to FAAH found in mature adipocytes in this study. The effects of MGL activity on 2-AG signalling are substantial, as demonstrated recently in mouse models, showing that both systemic MGL inhibition and MGL gene deletion lead to increased 2-AG levels in the brain and peripheral tissues, and desensitisation of brain CB_1 _receptors [35,36]. As MGL is not thought to be under hormonal control in triglyceride catabolism, it has not been extensively investigated in relation to obesity. However, given the importance of MGL in 2-AG signalling in the ECS, and considering that plasma 2-AG rises with obesity, we investigated whether MGL activity changes with BMI or other markers of adiposity. In contrast to our observations with FAAH activity, we found that MGL activity in subcutaneous adipocytes has no relationship with BMI, adiposity, serum adipokine levels or glycaemic regulation. This is in agreement with studies showing that MGL mRNA levels in subcutaneous adipose tissue are not different between lean and obese humans [19] and intracellular 2-AG levels are increased in hypertrophic adipocytes [7]. More generally, our findings are in agreement with the observation that the rate of glycerol release from adipose tissue is the same in lean and obese subjects, in both fasting and fed states [37]. Our findings suggest that, with regard to adipocyte contribution to generalised 2-AG catabolism, the increase in circulating 2-AG observed in obese humans may be due to enhanced production rather than decreased degradation.

In conclusion, the results of this study provide novel evidence that FAAH activity, and thus the rate of endocannabinoid degradation, in human subcutaneous mature adipocytes from healthy humans increases with BMI and waist circumference, but not with other markers of adiposity or metabolism. Conversely, MGL activity does not correlate with BMI or any other markers measured in this study. These findings support the hypothesis that some components of the ECS are upregulated with increasing adiposity in humans.

## Competing interests

The authors declare that they have no competing interests.

## Authors' contributions

All authors participated in the review of the manuscript. JC took the anthropometric measurements, performed the assays, conducted the statistical analysis and drafted the manuscript. GT conceived of the study, participated in its design and obtained adipose tissue and blood samples from the volunteers. SA developed the enzyme assay design. SO conceived of the study and participated in its design. All authors read and approved the final manuscript.
